# Effects of Copper Chelation on BRAF^V600E^ Positive Colon Carcinoma Cells

**DOI:** 10.3390/cancers11050659

**Published:** 2019-05-12

**Authors:** Silvia Baldari, Giuliana Di Rocco, Marie C. Heffern, Timothy A. Su, Christopher J. Chang, Gabriele Toietta

**Affiliations:** 1 Department of Research, Advanced Diagnostic, and Technological Innovation, IRCCS Regina Elena National Cancer Institute, via E. Chianesi 53, 00144 Rome, Italy; silvia.baldari@ifo.gov.it (S.B.); giuliana.dirocco@ifo.gov.it (G.D.R.); 2 Department of Chemistry, University of California, Davis, One Shields Avenue, Davis, CA 95616, USA; mcheffern@ucdavis.edu; 3 Departments of Chemistry and Molecular and Cell Biology, University of California, Berkeley, CA 94720, USA; tim_su@berkeley.edu (T.A.S.); chrischang@berkeley.edu (C.J.C.); 4 Howard Hughes Medical Institute, University of California, Berkeley, CA 94720, USA

**Keywords:** copper, colon cancer, BRAF, tetrathiomolybdate, chelating agents, mitogen-activated protein kinase, BRAF^V600E^ mutation

## Abstract

High affinity copper binding to mitogen-activated protein kinase kinase 1 (MAP2K1, also known as MEK1) allosterically promotes the kinase activity of MEK1/2 on extracellular signal regulated kinases 1 and 2 (ERK1/2). Consequently, copper-dependent activation of the mitogen-activated (MAP) kinase pathway has a role in promoting tumor growth. Conversely, copper chelation may represent a possible therapeutic approach for a specific subset of tumors characterized by activating mutations in the serine/threonine protein kinase V-Raf Murine Sarcoma Viral Oncogene Homolog B1 (BRAF), such as the V600E, occurring within the kinase domain (BRAF^V600E^). Tetrathiomolybdate (TM) is a specific copper chelating agent currently used for the treatment of Wilson’s disease and in preclinical studies for the management of metastatic cancers owing to its anti-angiogenic and anti-inflammatory properties. We evaluated in vitro and in vivo the effects of copper depletion achieved by pharmacological treatment with TM in human colorectal cells bearing the BRAF^V600E^ mutation in comparison with BRAF wild type cells. We provide evidence that selective copper chelation differentially affects proliferation, survival and migration of colon cancer cells bearing the BRAF^V600E^ mutation compared to BRAF^wt^ acting via differential phosphorylation levels of ERK1/2. Moreover, tetrathiomolybdate treatment was also effective in reducing the clonogenic potential of colon cancer BRAF^V600E^ cells resistant to BRAF pharmacological inhibition. In conclusion, these results support further assessment of copper chelation therapy as an adjuvant therapy for inhibiting the progression of colon cancers containing the BRAF^V600E^ mutation.

## 1. Introduction

Colorectal cancer is one of the most common cancers and a leading cause of cancer-associated death. The relative survival rate 5-year following diagnosis is 65%, but it drops to 13% once the disease has spread [1]. A specific subset of colorectal cancer characterized by poor survival, prevalence in women in advanced age, location in the proximal colon, and microsatellite instability, is represented by the tumors bearing a mutation in the serine/threonine protein kinase V-Raf Murine Sarcoma Viral Oncogene Homolog B1 (BRAF) [2]. In particular, approximately 80%–90% of all BRAF mutations observed in colon cancer patients are represented by the missense mutation 1799T>A within the kinase domain of BRAF (BRAF c.1799T>A), which leads to the substitution of a valine to a glutamic acid at the position 600 (BRAF*^V600E^*). The BRAF^V600E^ mutation accounts for 10% of all colorectal cancers, 60% of melanomas, 40% of thyroid carcinomas and it has been observed with different prevalence also in other tumors [3,4]. Compared to its wild type counterpart, mutant BRAF^V600E^ has an increased phosphorylation activity on the mitogen-activated protein kinases 1 and 2 (MEK1 and MEK2), which in turn activate the extracellularly regulated kinases 1 and 2 (ERK1 and ERK2), thereby activating the mitogen-activated protein kinase (MAPK) pathway to promote cancer [5]. BRAF^V600E^ is a promising druggable target for therapy and several BRAF inhibitors such as vemurafenib and dabrafenib have been developed [6]. In particular, selective targeting using BRAF inhibitors is effective in most (up to 80%) BRAF^V600E^ mutant melanomas, but the promising results observed in melanoma monotherapy were not reproduced in colorectal cancer patients, with a response rate of approximately 5% [7,8]. Therefore, it is necessary to further investigate the different response mechanisms between the two types of cancer and to develop innovative strategies to suppress MAPK activity in BRAF-mutant colorectal cancer [7].

Interestingly, it has been demonstrated that copper (Cu) availability is critical for the kinase activity of MEK1 and MEK2. In particular, copper influx enhances MEK1 phosphorylation of ERK1 and ERK2 through a Cu–MEK1 interaction [9] promoting tumor growth. Consequently, reduced expression of the primary copper transporter (Cu transporter 1, CTR1), or mutations in MEK1 which disrupt Cu binding, decrease BRAF^V600E^-driven signaling and tumorigenesis [10]. In addition, patients with different type of tumors, including advanced colon cancer, show elevated serum copper concentration [11] with an increased mortality risk [12]. Moreover, patients with drug-resistant tumors have 130%–160% more copper in their serum than those with drug-sensitive ones [13]. Copper has also a role in promoting tumor angiogenesis [14]. Collectively, these observations argue that reduction of Cu levels through pharmacological treatment or lowering Cu dietary intake, might represent an innovative strategy to inhibit progression of cancers containing the BRAF^V600E^ mutation [13].

In the current manuscript, we evaluated the effects of Cu depletion achieved by pharmacological treatment with the specific chelating agent tetrathiomolybdate (TM), currently used for the treatment of Wilson’s disease [15], in BRAF^V600E^ colorectal cancer cells in vitro and in vivo.

## 2. Results

### 2.1. Copper Modulation Differentially Affects Proliferation, Survival and Migration of Colon Cancer Cells Bearing BRAF^V600E^ Mutation Compared to BRAF^wt^

**Figure 1:**
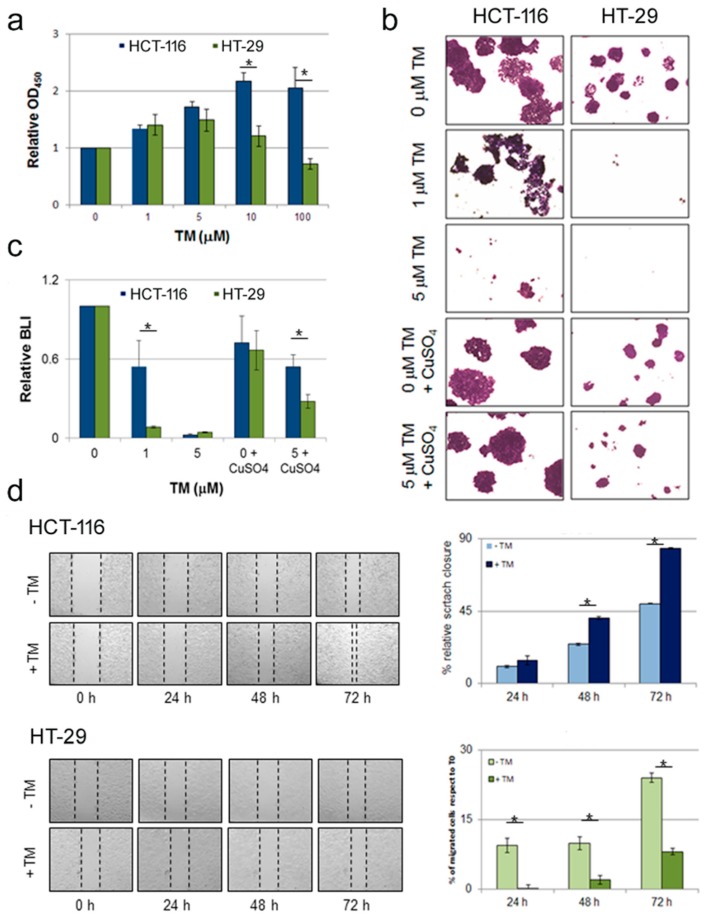
Copper chelation selectively affects proliferation, survival and migration of colon cancer HT-29 cells bearing BRAF^V600E^ mutation compared to HCT-116 cells carrying BRAF^wt^. **(a)** Copper depletion by treatment with the chelating agent tetrathiomolybdate (TM) affects short-term proliferation and survival of colon cancer cells bearing BRAF^V600E^ mutation. Colon cancer cell lines BRAF^wt^ (HCT-116) and BRAF^V600E^ (HT-29) were treated with TM (dose range 1-100 μM). After 24 hours the WST-1 cell proliferation assay was performed. Results are reported as means ± S.D. of three independent experiments. **(b)** TM treatment affects clonogenic ability of BRAF^V600E^ colon cancer cells. Long-term response to TM treatment was evaluated by clonogenic assay. BRAF^wt^ and BRAF^V600E^ colon cancer cells were seeded at a density of 1.5x10^3^/60-mm tissue culture dish and treated with TM, CuSO_4_ or both. Media were changed every 3–4 days. After 10 days of culture colonies were stained with crystal violet and then observed under an inverted microscope. Representative images are shown. The experiment was performed two times in duplicate. (**c**) TM treatment reduces bioluminescent emission from BRAF^V600E^ compared to BRAF^wt^ colon cancer cells expressing luciferase. Non-invasive bioluminescent analysis using D-luciferin as substrate performed on BRAF^wt^ and BRAF^V600E^ colon cancer cells engineered to express luciferase exposed to TM for 1 week. **(d)** TM treatment inhibits cells migration ability of BRAF^V600E^ colon cancer cells. Scratch assay was performed on colon cancer HCT-116 (upper panel) and HT-29 cells (lower panel). A 1-mm wide scratch was performed on cells monolayer, then cells were treated with 1 μM TM. Cells migration and scratch closure was assessed every 24 hours. Left panels show representative images taken at different time points. The scratched area was determined using the ImageJ software, and the quantitative data are shown in the right panels as mean ± S.D. of three independent experiments. In all panels * denotes *p* < 0.05.

We evaluated the impact of copper chelation on in vitro proliferation of two human colorectal carcinoma cell lines characterized by the presence of BRAF^wt^ (HCT-116) or carrying the BRAF^V600E^ mutation (HT-29) [3]. Cu depletion in the culture medium was achieved through pharmacological treatment with tetrathiomolybdate (TM), a highly specific copper chelator. Conversely, supplementation of copper was obtained through culture medium supplementation with increasing concentration of cupric sulfate (CuSO_4_). The effect of copper chelation on the viability of colon cancer cells bearing the wild type or mutant BRAF gene was determined using a colorimetric (WST-1 based) assay (Figure 1a). BRAF^wt^ cells (HCT-116) showed an increase in the proliferation rate, whereas the same treatment led to a significant reduction in cell survival of BRAF^V600E^ cells (HT-29) (Figure 1a), thus suggesting that BRAF^V600E^ cells are more sensitive to copper depletion.

Next we performed a clonogenic assay on colon cancer cells maintained in culture for ten days in presence of TM. As shown in Figure 1b, treatment with 1 µM TM drastically impacted on clonogenic cell survival of BRAF^V600E^ colon cancer HT-29 cells, with minimal effect on BRAF^wt^ HCT-116 cells. At increasing concentration (5 μM) a toxic effect was assessed in both cell lines. The reduced clonogenic ability in BRAF^V600E^ cell upon TM treatment was completely rescued by supplementation with cupric sulfate (50 mM CuSO_4_), indicating a specific prominent role for copper concentration in differential modulation of human colorectal carcinoma cells and indirectly confirming the specific copper chelation properties of TM.

As a more quantitative approach to assess the effect of copper chelation on human colorectal carcinoma cells, we cultured luciferase expressing BRAF^wt^ HCT-116 cells and BRAF^V600E^ HT-29 cells in the presence of 1 μM TM for a week and then performed a quantitative bioluminescence analysis. Efficient light emission results from luciferase-mediated oxidation of D-luciferin which requires Mg^2+^ and ATP, both provided by the cellular metabolism. Consequently, only living cells expressing luciferase are able to produce a signal detectable by bioluminescence imaging (BLI). Therefore, in this experimental setting, quantification of light emission can be considered a cell vitality assay surrogate. Compared to the corresponding cells cultured in complete medium without any supplementation, light produced by BRAF^wt^ cells after 1 week of culture in presence of 1 μM TM was slightly reduced, while emission in BRAF^V600E^ cells cultured in the same conditions were approximately 30% of the control (*p* < 0.05) (Figure 1c). As for the experiment described in Figure 1c, the anti-proliferative effect of TM treatment in BRAF^V600E^ cells was recovered by cupric sulfate supplementation, while supplementation with CuSO_4_ alone did alter BLI imaging significantly. 

In addition, we used BLI as a surrogate indication for determining the effect of TM treatment on copper cellular content. To this extent we used the Copper-Caged Luciferin-1 (CCL-1), a bioluminescent reporter synthesized for in vivo copper visualization by bioluminescence [16]. In both luciferase expressing BRAF^wt^ HCT-116 cells and BRAF^V600E^ HT-29 cells, treatment with TM induced a significant reduction on bioluminescence, compared to relative cells cultured in medium not supplemented with TM. BLI analysis performed on cells cultured in the same conditions and incubated with firefly luciferase did not show any significant difference. This result suggests that TM supplementation results in a similar reduction of cellular copper content in both BRAF^wt^ and BRAF^V600E^ colon cancer cells.

To gain further insights into the in vitro effect of copper chelation treatment on colon cancer cells with a different status in BRAF, we performed a scratch assay [17] to evaluate the effect of pharmacological copper chelation on disrupted monolayers of HCT-116 and HT-29 cell lines. The assay was performed in low serum concentration (serum starvation) to minimize the effect of cell proliferation. As shown in Figure 1d, both cell lines were able to migrate through the scratched area. However, while BRAF^wt^ HCT-116 cells treated with TM required a shorter time to close the wound compared to the corresponding untreated points (Figure 1d, upper panel), scratch healing was significantly inhibited in BRAF^V600E^ HT-29 cell lines under the same treatment (Figure 1d, lower panel), thus suggesting that upon copper chelation BRAF^V600E^ colon cancer cell migration ability is reduced.

Collectively, these data support the hypothesis that the effects of pharmacological copper chelation on colon cancer cell lines are cell context dependent; in particular, copper depletion results in a reduction of both cell proliferation rate and migration ability in BRAF^V600E^ HT-29 mutant colon cancer cells, while the same treatment on BRAF^wt^ HCT-116 colon cancer cells does not have a detrimental effect on proliferation.

### 2.2. Copper Modulation Differentially Affects Phosphorylation of ERK1/2 in BRAF^wt^ and BRAF^V600E^ Colon Cancer Cells Lines

**Figure 2:**
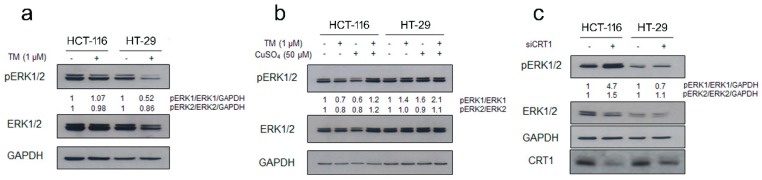
Copper chelation selectively affects MAPK signaling modulation in BRAF^wt^ and BRAF^V600E^ colon cancer cells. (**a**) BRAF^wt^ (HCT-116) and BRAF^V600E^ (HT-29) (1.5 × 10^5^ cells/60 mm dish) were treated with the chelating agent TM (1 *μ*M) for 72 hours. Immunoblot analysis was performed on cell lysates to assess the phosphorylation state of ERK1/2. Densitometry was performed using ImageJ software. Relative values of pERK1/2 band intensities of TM-treated points were normalized to the corresponding total protein form (ERK1/2) and to GAPDH (loading control) and finally quantified with respect to untreated control, arbitrarily set to 1.0. (**b)** Copper sulfate treatment rescues pERK decrease in BRAF^wt^ colon cancer cells upon TM treatment. BRAF^wt^ and BRAF^V600E^ were treated either with TM (1 μM), with CuSO_4_ (50 μM) or with a combination of both. After 72 hours of treatment cells were collected to assess the phosphorylation state of ERK1/2 by immunoblot using specific anti-ERK1/2 (phosphorylated and total form) and anti-GAPDH antibodies. (**c**) Knockdown of CTR1, as pharmacological copper depletion with TM, decreases MAPK signaling in BRAF mutant colon cancer cell lines. CRT1 silencing by small interfering RNA was performed and 48 hours later cells were harvested to assess the phosphorylation state of ERK1/2 by immunoblot.

BRAF^V600E^ is referred to as class I mutant [4], a group of BRAF mutations giving rise to stronger activation of BRAF kinase activity and constitutive activation of the MAPK pathway compared to the wild type counterpart [5]. Intriguingly, MEK1 phosphorylation of ERK1/2 is enhanced by copper availability [9]. As a result, BRAF^V600E^-driven signaling and tumorigenesis are reduced either by MEK1 mutations which disrupt Cu binding or decreased cellular copper uptake [10]. In the light of these considerations, we assayed the ability of TM treatment to influence signal transduction through the Raf1/MEK/ERK pathway in colon cancer cells bearing different status of BRAF. In particular, by immunoblot analysis we examined the phosphorylation status of ERK1/2, the mitogen-activated protein kinases involved in the signal transduction cascade that leads to cell proliferation, in cells treated with TM. The level of phosphorylated ERK1/2 kinases in BRAF^V600E^ colon cancer HT-29 cells decreased significantly during incubation with TM (Figure 2a). Moreover, a slight reduction of total ERK proteins was also determined in the same cell line. In contrast, no reduction was observed under copper chelation treatment in BRAF^wt^ cells (Figure 2a). Conversely, copper supplementation in HT-29 cells treated with TM rescued the pERK1/2 decrease observed upon TM treatment alone, while a further increase in the phosphorylation level of ERK1/2 was observed in HCT-116 cells (Figure 2b). We further investigated the role of cellular copper content in BRAF^V600E^ driven tumorigenesis by reducing the Cu influx through knockdown of the high-affinity Cu-transporter CRT1 (Figure 2c). In accordance with the effects observed upon pharmacological copper chelation by TM, CRT1 silencing could also affect MAPK pathway activation. The BRAF^V600E^ cells with reduced expression of CRT1 exhibited reduced phosphorylation of ERK1/2 (P-ERK1/2), while robust activation was induced in HCT-116 cells under the same experimental conditions. Thus, BRAF^V600E^ requires the copper transport function of CTR1 for increased signaling and tumorigenesis.

### 2.3. Copper Chelation Differently Affects E-Cadherin Expression in BRAF^wt^ and BRAF^V600E^ Colon Cancer Cell Lines

**Figure 3:**
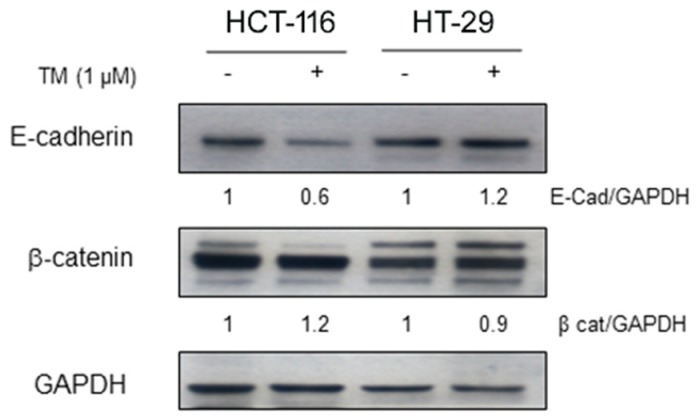
Copper chelation differently affects E-cadherin expression of colon cancer HT-29 cells bearing BRAF^V600E^ mutation compared to HCT-116 cells carrying BRAF^wt^**.** HCT-116 and HT-29 were treated with TM (1 μM) for 72 hours and then collected to evaluate specific anti- E-cadherin and β-catenin expression by immunoblot. Anti-GAPDH was used as loading control. Densitometry was performed using ImageJ software.

Modulation of the adhesion protein E-cadherin has been associated to the progression of various epithelial tumors. To assess whether copper chelation could affect E-cadherin expression in BRAF^V600E^ mutant cells and in their wild type counterpart, HT-29 and HCT-116 colon cancer cell lines were treated with TM (1 µM), the same concentration at which we observed pERK1/2 modulation. After 72 hours, TM significantly decreased the expression of E-cadherin in HCT-116 cells whereas an increase was observed in HT-29 cells (Figure 3), thus suggesting that the increased proliferation rate of HCT-116 cells treated with TM could be associated with a more aggressive tumor phenotype and increased tumor cell invasiveness. Conversely, no changes were revealed on the expression of the adhesion molecule β-catenin in both cell lines.

### 2.4. Copper Modulation Differentially Affects Proliferation of BRAF^wt^ and BRAF^V600E^ Colon Cancer Cells in a Three-Dimensional Tumor Spheroid Model

**Figure 4:**
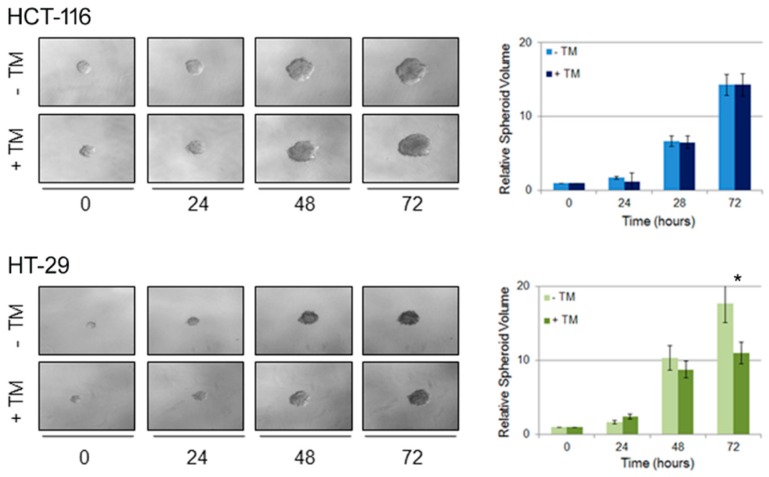
Copper chelation differently affects 3D proliferation of tumor colon cancer spheroids bearing the BRAF^V600E^ mutation. Colon cancer cell lines BRAF^wt^ (HCT-116) and BRAF^V600E^ (HT-29) were seeded on ultra-low attachment dish allowing for tumor spheroid formation. Four days later, spheroids were embedded into Matrigel and then treated with TM 10 μM. Tumor spheroid formation was assessed at different time points thereafter and spheroids volume was quantified according to the formula: V = a × (b^2^)/2, where a and b are spheroid length and width, respectively. Representative images of spheroids derived from both cell lines are shown in the left panels. Spheroid volume are reported on the right panels. Results are expressed as means ± S.D. of three independent experiments. * (*p* < 0.05) TM-treated cells values with respect to untreated cells.

In order to better characterize the effect of copper depletion on colon cancer cells bearing wild type or mutant BRAF, we performed a three-dimensional (3D) culture spheroid proliferation assay [18]. Spheroids are three-dimensional structures composed of cancer cells aggregated in vitro which can mimic tumor behavior more effectively than conventional two-dimensional (2D) cell cultures because of their mixed composition [19]. They can in fact contain both surface-exposed and deeply buried cells, proliferating and quiescent cells, well-oxygenated and hypoxic cells [20], thus better recapitulating the in vivo tissue microenvironment. To achieve this aim, HCT-116 and HT-29 colon cancer cells were seeded in ultra-low attachment cell culture dish; then spheroids were embedded into Matrigel and subsequently treated with TM. Spheroids proliferation derived from both cell lines was recorded acquiring images every 24 hours. The volume of the spheroids treated with TM was compared to the volume of spheroids cultured in control medium (Figure 4). TM treatment induced differential effects on the proliferation rate: in BRAF^wt^ HCT-116 cells copper chelation induced a higher proliferation rate compared to the corresponding untreated point, whereas HT-29 BRAF^V600E^ cells treated with TM proliferated less than untreated cells. The results obtained with the 3D spheroid tumor model are in agreement with the data obtained by (2D) culture assay, clearly showing that copper depletion negatively impacts on BRAF^V600E^ colon cancer cell proliferation.

### 2.5. Copper Chelation Therapy Affects Human Tumor Growth in a Xenograft Model In Vivo

**Figure 5:**
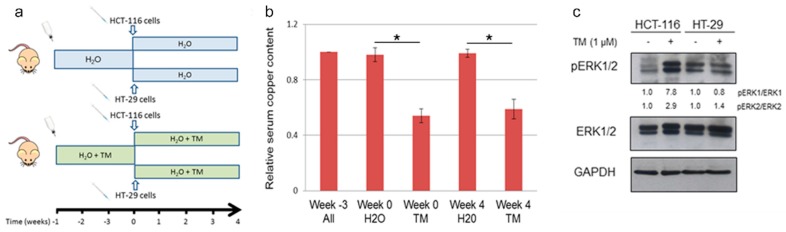
Copper chelation results in reduction of serum copper content and in modulation of MAPK signaling in an in vivo human colon carcinoma xenograft model. (a) Schematic representation of animal study: animals were randomly assigned to two group (8 mice each), one receiving drinking water and the second drinking water supplemented with TM. After 3 weeks each group was divided in two subgroups: one receiving subcutaneous administration of HCT-116-luc and the second HT-29-luc. Tetrathiomolybdate administration effectively reduced serum copper content (**b**) and modulated ERK1/2 phosphorylation in a xenograft model of BRAF^V600E^-derived colorectal tumors, as assessed by immunoblot analysis (**c**). * (*p* < 0.05).

To validate in vivo the results obtained in vitro, we performed a pilot study to evaluate the effect of copper chelation therapy in a xenograft model of human tumor growth (Figure 5a). Immunodeficient mice were randomly assigned to two experimental groups: the first group received ad libitum drinking water without any supplementation; the second group received drinking water supplemented with TM throughout the entire duration of the experiment [21]. The effect of TM treatment in reducing serum copper content was confirmed by the determination of copper in blood drawn from mice in both groups at different time points (Figure 5b). Three weeks after the beginning of TM supplementation, BRAF^wt^ (HCT-116) or BRAF^V600E^ (HT-29) colon cancer cells stably expressing luciferase were mixed with Matrigel and subcutaneously injected into mice in each group. BLI analysis was performed 1 day after tumor cell inoculation, revealing a similar bioluminescence signal in correspondence of the site of injection in both groups. Then tumor progression was assessed weekly by caliper measurement and BLI imaging until tumor size reached the dimension requiring humane intervention. Bioluminescent signal intensity increased over the course of several weeks, correlating with increased caliper measurement in all experimental groups. Tumor progression was similar in animal receiving BRAF^wt^ cells regardless of whether there was TM supplementation in the drinking water. Also, differences observed in dimension and BLI emission of tumors derived from inoculation of BRAF^V600E^ cells in animals whether they were receiving TM supplementation or not failed to reach statistical significance (*p* = 0.083), although animals that received TM had decreased tumor size and BLI emission compared to controls 1 month after receiving HT-29 cells.

At necropsy, tumors were excised. The effect of systemic copper depletion on the phosphorylation state of ERK1/2 was determined by immunoblot analysis performed on tumor lysates. In agreement with the experiments performed on cultured cells in vitro, we also observed in tumor tissue derived from cells inoculated in vivo a similar reduction in the ratio of phospho/total ERK1/2 in HT-29 TM treated specimens, whereas in HCT-116 this ratio was increased as in the in vitro assays (Figure 5c).

Collectively these data confirm the use of TM treatment in reducing serum copper levels. However, in the current experimental setting no statistical significant difference was detectable amongst the different groups, suggesting that the copper chelation treatment does not affect the size of primary tumors deriving from both BRAF^wt^ and BRAF^V600E^ colorectal cells. Nonetheless, we were able to assess that TM supplementation resulted in modulation of ERK1/2 phosphorylation in tumor cells in a xenograft model of BRAF^V600E^-derived colorectal tumors.

### 2.6. Effect of TM Treatment on BRAF Inhibitor Resistant Cells

**Figure 6:**
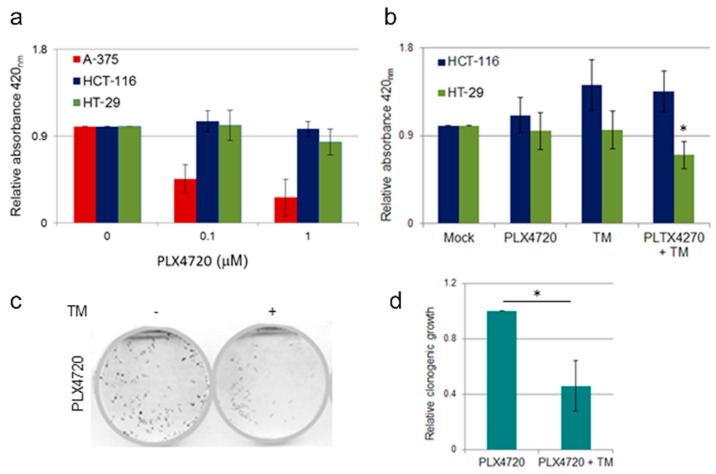
Effect of TM treatment on BRAF inhibitor resistant cells. (**a**) Melanoma and colon cancer cell lines show different sensitivity to PLX4720 treatment. Melanoma cell line A375 (BRAF^V600E^), colon cancer HCT-116 (BRAF^wt^) and HT-29 (BRAF^V600E^) were treated with the increasing doses of PLX4720. After 48 hours of treatment, cell survival was quantified using the WST-1 cell proliferation assay kit. (**b)** Combination therapy of PLX4720 and TM affects BRAF^V600E^ colon cancer cell proliferation. HCT-116 (BRAF^wt^) and HT-29 (BRAF^V600E^) were treated with PLX4720 1 µM or TM 10 µM alone or in combination. After 24 hours of treatment, cell survival was quantified using the WST-1 cell proliferation assay kit. **c)** TM treatment affects clonogenic ability of BRAF mutant colon cancer cells resistant to PLX4720. PLX4720-resistant colon cancer HT-29 cells were established by continuous exposure to step-wise increasing concentrations of PLX4720, from 0.1 µM to 4 µM, until the surviving cells reached 90% confluence. Response to combination treatment with PLX4720 and TM was evaluated by clonogenic assay in HT-29-PLX4720- resistant cells. The medium was changed every 3–4 days and after 10 days grown colonies were stained with crystal violet (**c**) and quantified by densitometric analysis (**d**). Results are reported as means ± standard deviation of three independent experiments. * *p* < 0.05.

Inhibition of the BRAF oncoprotein by vemurafenib has been shown to be highly effective in BRAF^V600E^ melanoma patients [22]; in contrast, the same treatment has limited effect on BRAF^V600E^ colon cancer patients due to intrinsic resistance against BRAF inhibitors [6,23]. We investigated whether copper chelation in combination with chemotherapy could improve the therapeutic response to vemurafenib. To achieve this aim, we analyzed cell survival and proliferation rates in response to increasing doses of the vemurafenib structural analogue PLX4720 in both BRAF^wt^ and BRAF^V600E^ colon cancer cells. A BRAF^V600E^ melanoma cell line, A-375, was used as an internal control to verify the efficiency of PLX4720 treatment. As expected PLX4720 treatment had no effect on cell proliferation in the BRAF^wt^ colon cancer cell line HCT-116, a modest effect on BRAF^V600E^ colon cancer cell line HT-29 at the highest tested dose, whereas it significantly impacted on survival of mutant BRAF^V600E^ melanoma A-375 cells (Figure 6a). We then tested the effect on cell proliferation of a combination therapy of PLX4720 and copper chelating agent TM. To this aim, HCT-116 and HT-29 colon cancer cell lines were challenged with 1 µM PLX4720 and 10 µM TM, alone or in combination (Figure 6b). We observed that in HT-29 cell line, a combination treatment of TM and PLX4720 was able to induce a decrease in cell proliferation with respect to single treatments, suggesting that copper depletion might confer sensitivity to BRAF inhibition by PLX4720 in colon cancer cells bearing BRAF^V600E^.

In the attempt to overcome the occurrence of acquired resistance in mutant BRAF cells to PLX4720 monotherapy, we tested the effect of copper chelating agent TM on HT-29 cells resistant to PLX4720. Cells resistant to PLX4720 were established as previously described [23] and clonogenic assay was performed. As shown in Figure 6c, the reduced clonogenic cell survival of HT-29 cells upon PLX4720 treatment was markedly improved by copper chelation, compared to cells treated by PLX4720 alone, as confirmed by densitometric analyses of clonogenic assays (Figure 6d). These findings reveal a potential additive effect of copper depletion and BRAF inhibition, thus suggesting that copper chelation therapy combined with pharmacological treatment with PLX4720 would provide a better therapeutic response in BRAF^V600E^ mutant colorectal cancer cells, which were otherwise unresponsive to vemurafenib or its analogue compounds treatments.

## 3. Discussion

Repurposing or repositioning copper chelating agents for cancer therapy is gaining increasing attention [24,25,26]. Copper, either acting through direct or indirect mechanisms, influences cancer progression, metastasis and induction of resistance to current therapies [12,13]. In particular, copper is an important angiogenic cofactor [14,27]; as a redox-active metal, copper can induce alterations in the redox status of cancer cells [28] and act as a tumor promoter [29]; copper depletion induces proteasome inhibition and apoptosis in cancer cells [30]; moreover, copper may promote tumorigenesis via activation of the oncogenic MAPK pathway [10].

The role of copper in the modulation of BRAF signaling is particularly relevant in colon cancer [31]. The BRAF^V600E^ mutation, which renders BRAF constitutively active with a 500-fold increase in kinase activity compared to wild-type BRAF [5], represents approximately 90% of all BRAF mutations observed in colon cancer patients. Treatment with BRAF inhibitors is effective in approximately 50% of melanoma patients bearing the BRAF^V600E^ mutation, providing a significant survival benefit, even if acquired resistance can occur. In contrast, 95% of BRAF^V600E^ mutant colon cancer patients do not respond to BRAF inhibition monotherapy [4,32]. Thus, a critical unmet need exists for alternative strategies to repress MAPK activity in BRAF^V600E^ colorectal cancer patients [8]. It has been estimated that colon cancer cells accumulate up to seven fold higher copper levels than normal colon cells [33]. Moreover, levels of serum copper [34] and ceruloplasmin, which binds approximately 95% of the copper in the plasma [35], are significantly increased in patients with colorectal cancer compared to healthy controls. Increment in serum copper levels may be detected several years before cancer diagnosis [36] and correlates with tumor progression [37]. In addition, transcriptome analysis in colorectal cancer samples and cell lines revealed a marked upregulation of the specific primary copper transporter CTR1 and altered expression of other genes correlated to copper homeostasis [38]. Consequently, copper chelation therapy may selectively target cancer cells, having little or no toxicity on normal cells [28].

Copper chelating drugs have been evaluated in cancer therapy on the account of their anti-angiogenic properties [13,39,40,41]. In particular, tetrathiomolybdate (TM), a widely available drug developed for the treatment of Wilson’s disease, has been used as either an adjuvant or as primary therapy in several clinical trials for metastatic cancers such as breast [42,43], colorectal [44], kidney [45], prostate [46], lung [47], and esophageal cancers [48]. Collectively, these trials proved that TM treatment is well tolerated, with no apparent toxicity [49,50]. Overall, TM therapy, albeit while minimally affecting the primary tumor size, seems to prevent further tumor growth and metastatic spreading [27]. 

The discovery of the involvement of copper in stimulating the RAS-RAF-MEK-ERK (MAPK) signaling pathway has provided the bases for the use of chelating therapy in a subset of tumors characterized by BRAF activating mutations [51]. Specifically, copper binding to MEK1/2 allosterically promotes the kinase activity of MEK1/2 on ERK1/2 [10]. Copper depletion can reduce human melanoma BRAF^V600E^ cells growth [10,52]. Accordingly, our results indicate that copper chelation by TM treatment has a cytostatic effect also on proliferation of colorectal cancer cells bearing the BRAF^V600E^ mutation via inhibition of the MAPK cascade. In particular, we provide evidence obtained using different approaches such as 2D and 3D in vitro tumor growth and preliminary results in an in vivo xenograft model of colon cancer. In particular, we show that copper modulation differentially affects proliferation, survival and migration of colon cancer in HT-29 cells compared to HCT-116 BRAF^wt^ cells acting via differential phosphorylation levels of ERK1/2. Colorectal cancers cells used in the study have a different mutation status in several critical genes involved in colorectal cancer in addition to BRAF (HCT-116 wt; HT-29 V600E) including TP53 (HCT- 116 wt; HT-29 mutation R273H), KRAS (HCT-116 mutation G13D; HT-29 wt), PIK3CA (HCT-116 H1047R; HT-29 wt) [53]. Based on literature data [10,52] we assume that BRAF^V600E^ mutation may play a key role in the effects we observed, but due to the complexity of the differences of the mutational spectrum, other concomitant causes cannot be excluded. We also observed that TM treatment is effective in reducing clonogenic growth of colon cancer HT-29 BRAF^V600E^ cells resistant to BRAF pharmacological inhibition. Interestingly, copper plays a central role in drug resistance [11,54]. In particular, colorectal cancer patients responding and not responding to chemotherapy have respectively 110% and 171% more serum copper than healthy controls. Different mechanisms of resistance have been described, but reactivation of MEK signaling is considered to be a major determinant in the development of resistance to BRAF [55]. A combination of BRAF and MEK1/2 inhibition can overcome acquired resistance with improved response rates and patient progression-free survival in melanoma BRAF^V600E^ patients compared to BRAF monotherapy [56]. In addition, it has been previously suggested that TM treatment counters MAPK-dependent forms of resistance and improves the durability of the response to MAPK inhibitors in melanoma cells [52]. Our data suggest that TM treatment might also be effective on colon cancer BRAF^V600E^ cells which are resistant to BRAF pharmacological inhibition.

In conclusion, copper chelation therapy, which has been used for Wilson’s disease and for the management of metastatic cancers and suggested for the treatment of BRAF^V600E^ melanomas [51,52], can possibly be proposed as adjuvant therapy in BRAF^V600E^ colon cancer patients.

## 4. Materials and Methods

### 4.1. Cell Lines

Human colorectal carcinoma cell lines HCT 116 (ATCC CCL-347), HT-29 (ATCC HTB-38), firefly luciferase expressing HCT 116-luc (Caliper Life Sciences, Inc., Hopkinton, MA, USA) and HT-29-luc (Caliper Life Sciences) were cultured in Dulbecco’s modified Eagle’s medium (DMEM, Gibco, Grand Island, NY, USA) supplemented with 10% (v/v) fetal bovine serum (FBS) and 1% (v/v) penicillin–streptomycin solution (50 U/mL penicillin and 50 μg/mL streptomycin), in a humidified atmosphere of 95% air and 5% CO_2_ at 37 °C. HCT-116 cells are characterized by the presence of wild type BRAF (BRAF^wt^), whereas HT-29 cells are heterozygous for the BRAF^V600E^ mutation [3,57]. Human melanoma cell line A-375 homozygous for BRAF^V600E^ (ATCC CRL-1619) were cultured according to the American Type Cell Collection specifications [58]. Information on the cell lines used in the study, including mutation analysis, has been reported in Catalogue of Somatic Mutations in Cancer (COSMIC)- Cell Line Project [59,60,61].

### 4.2. Induction of BRAF Inhibition Resistance

Human colorectal carcinoma cells resistant to the BRAF inhibitor PLX4720 were established as previously described [23]. Briefly, HT-29 cells were continuously treated with step-wise increasing concentrations of PLX4720 (Selleck Chemicals, Houston, TX, USA), from 0.1 µM to 4.0 µM, until the surviving cells reached 90% confluence. Then, one resistant PLX4720-resistant HT-29 clone was isolated.

### 4.3. Small Interfering RNA Transfection Procedure

Small interfering-mediated knockdown into human colorectal carcinoma cell lines of the high affinity copper uptake protein 1 (CRT1) was achieved by siRNA transfection of sequences specifically targeting CRT1 as previously described [10]. HCT-116 and HT-29 cell lines were seeded into a 60-mm tissue culture dish at a density of 1.5 x 10^5^ and then transfected using the Lipofectamine RNAiMAX Transfection Reagent (Invitrogen, ThermoFisher Scientific, Waltham, MA, USA), according to the manufacturer’s instructions. HCT-116 and HT-29 CTR1-silenced cells were harvested 48 hours after transfection. 

### 4.4. In vitro Treatments and Cell Proliferation Assay

Human colorectal carcinoma cell culture medium was supplemented with the highly specific copper chelator ammonium tetrathiomolybdate (TM) (Sigma-Aldrich, St. Louis, MO, USA) (final concentration range: 0.01–10 μM) or cupric sulfate (CuSO_4_) (BDH Chemicals, Poole, UK) (final concentration range: 25–200 μM). Cell proliferation after treatment was measured using the WST-1 cell proliferation assay kit (Takara, Clontech, Mountain View, CA, USA), according to the manufacturer’s instructions. The optical density at 450 nm was assessed using a microplate reader (BioRad Laboratories Inc. Hercules, CA, USA). All experiments were performed at least two times in duplicate, and the relative cell viability (%) was expressed as a percentage relative to the untreated control cells.

### 4.5. Colony Formation Assay

Cells were plated at a density of 5.0 × 10^3^/60-mm tissue culture dish and then cultured in a humidified CO_2_ incubator (5% CO_2_/95% air) at 37 °C. Cells were then treated as reported, with TM, CuSO_4_ or PLX4720, with medium replenished every 3–4 days. After 10 days of culture, cells were stained with crystal violet solution (0.25% crystal violet, 10% formaldehyde, 80% methanol) and observed under an inverted microscope or analyzed by densitometry with ImageJ software [62]. Experiments were performed in duplicate and repeated twice.

### 4.6. In vitro Scratch Assay

Cellular migration was assessed by an in vitro scratch wound healing assay [17]. HCT-116 and HT-29 cells were seeded in six-well plates and incubated until they were 90% confluent. Cell monolayers were scratched using a pipette tip to produce an approximately 1-mm wide scratch; cells were then washed with PBS to remove cellular debris. Subsequently, cells were incubated in serum-free medium or in serum-free medium supplemented with TM (1 μM). Immediately after the scratch and for the three consecutive days, images were captured with a Zeiss Axiocam digital camera on a Zeiss Axioskop microscope equipped with a 20× objective (Carl Zeiss, Oberkochen, Germany). The distances between the scratch edges were measured by analysis of the digital images using the ImageJ software (National Institutes of Health, Bethesda, MD, USA). The experiments were performed in duplicate.

### 4.7. Immunoblotting

For Western Blot analysis, cells (1.5 × 10^5^ cells/60 mm dishes) were treated with TM or CuSO_4_ and then washed in PBS, harvested by scraping and lysed in 1x RIPA buffer (150 mM NaCl, 1% Triton X-100, 0.25% sodium deoxycholate, 0.1% SDS, 50 mM Tris/HCl, pH 8.0, and 20 mM EDTA) supplemented with 1× complete protease inhibitor cocktail (Sigma-Aldrich), 1 mM phenylmethylsulfonyl fluoride (Sigma-Aldrich), 50 mM sodium fluoride (Sigma), and 50 mM dithiothreitol (Bio-Rad, Hercules, CA, USA). Protein lysates (30 μg/lane) were analyzed on a 10% SDS–polyacrylamide gel. Immunoblot analysis was performed according to established protocols and filters were immuno-reacted with the following antibodies: rabbit polyclonal anti-p44/42 MAPK (Erk1/2); rabbit polyclonal anti-p44/42 MAPK (Erk1/2) (Thr202/Tyr204); rabbit monoclonal anti-E Cadherin; rabbit monoclonal anti- *β* Catenin (Cell Signaling Technology, Danvers, MA, USA). Glyceraldehyde phosphate dehydrogenase (GAPDH) was used as a protein loading control. Secondary HRP-conjugated anti-mouse or anti-rabbit (Bio-Rad) antibodies were used. Detection of immuno-reactions was performed by ECL kit (Amersham Biosciences, Glattbrugg, Switzerland). Densitometry analysis was performed using the ImageJ software.

### 4.8. Experimental Animal Procedures

The Regina Elena National Cancer Institute Animal Care and Use Committee reviewed the procedures involving mice according to the Guidelines of the National Institutes of Health and current National legislation (D.lgs 26/2014, 4 March 2014) [63].

The animals used in the study were 6-8 weeks old male nu/nu athymic nude mice (Charles River, Calco, Italy) housed in individual ventilated cages in a facility with constant temperature and a 12 hours light cycle. Mice were fed a standard diet (Mucedola, Settimo Milanese, Italy) with copper content of 6 mg/kg. Mice were randomly assigned to 2 experimental groups of 4 animals each: the first group received drinking water without any supplementation; in the second group TM (0.02 mg/mL) [21,22,23,24,25,26,27,28,29,30,31,32,33,34,35,36,37,38,39,40,41,42,43,44,45,46,47,48,49,50,51,52,53,54,55,56,57,58,59,60,61,62,63,64] was administered in the drinking water that was prepared fresh twice a week. Treatments began 3 weeks before tumor cells implantation [65].

Colorectal carcinoma cells expressing luciferase (0.5 × 10^6^ cells/mouse) mixed with Matrigel (Becton Dickinson, Franklin Lakes, NJ, USA) were implanted subcutaneously into the dorsal region of the animals. Heterotopic tumor formation was assessed using a caliper once a week and tumor volumes (TV) were estimated by the formula: TV=*a* × (*b*^2^)/2, where *a* is the tumor larger diameter (length) and *b* the corresponding perpendicular diameter (width). Blood was collected by retro orbital bleeding and serum copper content was evaluated at different timepoints during the procedure using the Copper Assay Kit (Sigma-Aldrich), according to the manufacturer’s protocol. At necropsy, tumor samples were harvested and lysed for Western blot analysis.

### 4.9. Optical Bioluminescence Imaging

In addition to caliper assessment, tumor progression was followed also by in vivo bioluminescence imaging (BLI) analysis performed once a week using the IVIS Lumina II equipped with the Living Image software for data quantification (PerkinElmer, Waltham, MA, USA), as previously described [66].

### 4.10. 3D Tumor Spheroids Invasion Assay

To generate 3D spheroids [18], HCT-116 and HT-29 colon cancer cells were diluted to obtain a cell density of 1.0 × 10^4^/mL or 0.5 × 10^4^/mL, respectively, then 200 µL/well were seeded into ultra-low attachment cell culture dish (96 well). Four days after seeding, spheroids (approximate size 300–500 µm) were formed and then embedded into Matrigel. To this aim, 100 µL of growth medium were removed and replaced with 100 µL of Matrigel on maintained on ice, then incubated at 37 °C to allow the Matrigel to solidify. After 1 hour, 100 µL of fresh medium were added and spheroids were then treated with TM for the indicated duration. Spheroids proliferation was followed with images captured every 24 hours. Spheroid volume was estimated by the formula: V = a × (b^2^)/2, where *a* and *b* are spheroid length and width, respectively.

### 4.11. Bioluminescence Analysis Using Copper Caged-Luciferin-1

Copper content of HCT-116 and HT-29 cells maintained in different experimental conditions, supplemented with TM or CuSO_4_ was further assessed by in vivo bioluminescence imaging using a specific Copper-Caged-Luciferin-1 (CCL-1) kindly provided by C.J. Chang [16]. Briefly, HCT-116 and HT-29 cells (2.5 × 10^3^ cells/96 multi well dishes) were supplemented with TM at different doses (5 or 50 µM) for 24 hours. Then the medium was removed and 100 µL of CCL-1 (75 µM) were added into the dishes. As control a parallel set of cells were incubated with firefly luciferase. The plates were imaged with the IVIS Lumina II equipped with the Living Image software as previously described, and the data was analyzed and quantified [67].

### 4.12. Statistical Analyses

Comparison between groups was performed using the INSTAT software (GraphPad, San Diego, CA, USA) using a two-tailed Student *t* test for unpaired data; the statistical significance level was set at *p* ≤ 0.05.

## 5. Conclusions

Copper chelation reduces proliferation, survival and migration of human colon cancer cells bearing the BRAF^V600E^ mutation compared to BRAF^wt^ cells, acting through inhibition of MEK1/2. Furthermore, in combination with the administration of BRAF inhibitors, copper chelation treatment results in a better therapeutic response in BRAF-mutant cells otherwise unresponsive to vemurafenib alone. These results support the potential use of copper chelation therapy in BRAF^V600E^-mutant colorectal cancers.

## References

[1] Stintzing S. (2014). Management of colorectal cancer. F1000Prime Rep..

[2] Clarke C.N., Kopetz E.S. (2015). BRAF mutant colorectal cancer as a distinct subset of colorectal cancer: Clinical characteristics, clinical behavior, and response to targeted therapies. J. Gastrointest. Oncol..

[3] Davies H., Bignell G.R., Cox C., Stephens P., Edkins S., Clegg S., Teague J., Woffendin H., Garnett M.J., Bottomley W. (2002). Mutations of the BRAF gene in human cancer. Nature.

[4] Dankner M., Rose A.A.N., Rajkumar S., Siegel P.M., Watson I.R. (2018). Classifying BRAF alterations in cancer: new rational therapeutic strategies for actionable mutations. Oncogene.

[5] Wan P.T., Garnett M.J., Roe S.M., Lee S., Niculescu-Duvaz D., Good V.M., Jones C.M., Marshall C.J., Springer C.J., Barford D. (2004). Mechanism of activation of the RAF-ERK signaling pathway by oncogenic mutations of B-RAF. Cell.

[6] Zhang W. (2015). BRAF inhibitors: The current and the future. Curr. Opin. Pharmacol..

[7] Corcoran R.B. (2015). New therapeutic strategies for BRAF mutant colorectal cancers. J. Gastrointest. Oncol..

[8] Ursem C., Atreya C.E., Van Loon K. (2018). Emerging treatment options for BRAF-mutant colorectal cancer. Gastrointest Cancer.

[9] Turski M.L., Brady D.C., Kim H.J., Kim B.E., Nose Y., Counter C.M., Winge D.R., Thiele D.J. (2012). A novel role for copper in Ras/mitogen-activated protein kinase signaling. Mol. Cell Biol..

[10] Brady D.C., Crowe M.S., Turski M.L., Hobbs G.A., Yao X., Chaikuad A., Knapp S., Xiao K., Campbell S.L., Thiele D.J. (2014). Copper is required for oncogenic BRAF signaling and tumorigenesis. Nature.

[11] Majumder S., Chatterjee S., Pal S., Biswas J., Efferth T., Choudhuri S.K. (2009). The role of copper in drug-resistant murine and human tumors. Biometals.

[12] Wu T., Sempos C.T., Freudenheim J.L., Muti P., Smit E. (2004). Serum iron, copper and zinc concentrations and risk of cancer mortality in US adults. Ann. Epidemiol..

[13] Denoyer D., Masaldan S., La Fontaine S., Cater M.A. (2015). Targeting copper in cancer therapy: ’Copper That Cancer’. Metallomics.

[14] Lowndes S.A., Harris A.L. (2005). The role of copper in tumour angiogenesis. J. Mammary Gland Biol. Neoplasia.

[15] Medici V., Sturniolo G.C. (2008). Tetrathiomolybdate, a copper chelator for the treatment of Wilson disease, pulmonary fibrosis and other indications. IDrugs.

[16] Heffern M.C., Park H.M., Au-Yeung H.Y., Van de Bittner G.C., Ackerman C.M., Stahl A., Chang C.J. (2016). In vivo bioluminescence imaging reveals copper deficiency in a murine model of nonalcoholic fatty liver disease. Proc. Natl. Acad. Sci. USA.

[17] Liang C.C., Park A.Y., Guan J.L. (2007). In vitro scratch assay: a convenient and inexpensive method for analysis of cell migration in vitro. Nat. Protoc..

[18] Vinci M., Gowan S., Boxall F., Patterson L., Zimmermann M., Court W., Lomas C., Mendiola M., Hardisson D., Eccles S.A. (2012). Advances in establishment and analysis of three-dimensional tumor spheroid-based functional assays for target validation and drug evaluation. BMC Biol..

[19] Weiswald L.B., Bellet D., Dangles-Marie V. (2015). Spherical cancer models in tumor biology. Neoplasia.

[20] Frieboes H.B., Zheng X., Sun C.H., Tromberg B., Gatenby R., Cristini V. (2006). An integrated computational/experimental model of tumor invasion. Cancer Res..

[21] Zeng C., Hou G., Dick R., Brewer G.J. (2008). Tetrathiomolybdate is partially protective against hyperglycemia in rodent models of diabetes. Exp. Biol. Med. (Maywood).

[22] Chapman P.B., Hauschild A., Robert C., Haanen J.B., Ascierto P., Larkin J., Dummer R., Garbe C., Testori A., Maio M. (2011). Improved survival with vemurafenib in melanoma with BRAF V600E mutation. N. Engl. J. Med..

[23] Mao M., Tian F., Mariadason J.M., Tsao C.C., Lemos R., Dayyani F., Gopal Y.N., Jiang Z.Q., Wistuba I.I., Tang X.M. (2013). Resistance to BRAF inhibition in BRAF-mutant colon cancer can be overcome with PI3K inhibition or demethylating agents. Clin. Cancer. Res..

[24] Denoyer D., Clatworthy S.A.S., Cater M.A. (2018). Copper complexes in cancer therapy. Met. Ions. Life Sci..

[25] Dou Q.P. (2012). Repositioning the old, existing copper-binding drugs for cancer treatment. Clin. Exp. Pharmacol..

[26] Wang F., Jiao P., Qi M., Frezza M., Dou Q.P., Yan B. (2010). Turning tumor-promoting copper into an anti-cancer weapon via high-throughput chemistry. Curr. Med. Chem..

[27] Goodman V.L., Brewer G.J., Merajver S.D. (2004). Copper deficiency as an anti-cancer strategy. Endocr. Relat. Cancer.

[28] Gupte A., Mumper R.J. (2009). Elevated copper and oxidative stress in cancer cells as a target for cancer treatment. Cancer Treat. Rev..

[29] Ishida S., Andreux P., Poitry-Yamate C., Auwerx J., Hanahan D. (2013). Bioavailable copper modulates oxidative phosphorylation and growth of tumors. Proc. Natl. Acad. Sci. USA.

[30] Daniel K.G., Chen D., Yan B., Dou Q.P. (2007). Copper-binding compounds as proteasome inhibitors and apoptosis inducers in human cancer. Front. Biosci..

[31] Barras D., Missiaglia E., Wirapati P., Sieber O.M., Jorissen R.N., Love C., Molloy P.L., Jones I.T., McLaughlin S., Gibbs P. (2016). BRAF V600E mutant colorectal cancer subtypes based on gene expression. Clin. Cancer Res..

[32] Kopetz S., Desai J., Chan E., Hecht J.R., O’Dwyer P.J., Maru D., Morris V., Janku F., Dasari A., Chung W. (2015). Phase II Pilot Study of Vemurafenib in Patients With Metastatic BRAF-Mutated Colorectal Cancer. J. Clin. Oncol..

[33] Fatfat M., Merhi R.A., Rahal O., Stoyanovsky D.A., Zaki A., Haidar H., Kagan V.E., Gali-Muhtasib H., Machaca K. (2014). Copper chelation selectively kills colon cancer cells through redox cycling and generation of reactive oxygen species. BMC Cancer.

[34] Gupta S.K., Shukla V.K., Vaidya M.P., Roy S.K., Gupta S. (1993). Serum and tissue trace elements in colorectal cancer. J. Surg. Oncol..

[35] Nayak S.B., Bhat V.R., Upadhyay D., Udupa S.L. (2003). Copper and ceruloplasmin status in serum of prostate and colon cancer patients. Indian J. Physiol. Pharmacol..

[36] Coates R.J., Weiss N.S., Daling J.R., Rettmer R.L., Warnick G.R. (1989). Cancer risk in relation to serum copper levels. Cancer Res..

[37] Linder M.C., Moor J.R., Wright K. (1981). Ceruloplasmin assays in diagnosis and treatment of human lung, breast, and gastrointestinal cancers. J. Natl. Cancer Inst..

[38] Barresi V., Trovato-Salinaro A., Spampinato G., Musso N., Castorina S., Rizzarelli E., Condorelli D.F. (2016). Transcriptome analysis of copper homeostasis genes reveals coordinated upregulation of SLC31A1,SCO1, and COX11 in colorectal cancer. FEBS Open Bio..

[39] Finney L., Vogt S., Fukai T., Glesne D. (2009). Copper and angiogenesis: Unravelling a relationship key to cancer progression. Clin. Exp. Pharmacol. Physiol..

[40] Antoniades V., Sioga A., Dietrich E.M., Meditskou S., Ekonomou L., Antoniades K. (2013). Is copper chelation an effective anti-angiogenic strategy for cancer treatment?. Med. Hypotheses.

[41] Goodman V.L., Brewer G.J., Merajver S.D. (2005). Control of copper status for cancer therapy. Curr. Cancer Drug Targets.

[42] Chan N., Willis A., Kornhauser N., Ward M.M., Lee S.B., Nackos E., Seo B.R., Chuang E., Cigler T., Moore A. (2017). Influencing the tumor microenvironment: A phase II study of copper depletion using tetrathiomolybdate in patients with breast cancer at high risk for recurrence and in preclinical models of lung metastases. Clin. Cancer Res..

[43] Jain S., Cohen J., Ward M.M., Kornhauser N., Chuang E., Cigler T., Moore A., Donovan D., Lam C., Cobham M.V. (2013). Tetrathiomolybdate-associated copper depletion decreases circulating endothelial progenitor cells in women with breast cancer at high risk of relapse. Ann. Oncol..

[44] Gartner E.M., Griffith K.A., Pan Q., Brewer G.J., Henja G.F., Merajver S.D., Zalupski M.M. (2009). A pilot trial of the anti-angiogenic copper lowering agent tetrathiomolybdate in combination with irinotecan, 5-flurouracil, and leucovorin for metastatic colorectal cancer. Invest. New Drugs.

[45] Redman B.G., Esper P., Pan Q., Dunn R.L., Hussain H.K., Chenevert T., Brewer G.J., Merajver S.D. (2003). Phase II trial of tetrathiomolybdate in patients with advanced kidney cancer. Clin. Cancer Res..

[46] Henry N.L., Dunn R., Merjaver S., Pan Q., Pienta K.J., Brewer G., Smith D.C. (2006). Phase II trial of copper depletion with tetrathiomolybdate as an antiangiogenesis strategy in patients with hormone-refractory prostate cancer. Oncology.

[47] Brewer G.J., Dick R.D., Grover D.K., LeClaire V., Tseng M., Wicha M., Pienta K., Redman B.G., Jahan T., Sondak V.K. (2000). Treatment of metastatic cancer with tetrathiomolybdate, an anticopper, antiangiogenic agent: Phase I study. Clin. Cancer Res..

[48] Schneider B.J., Lee J.S., Hayman J.A., Chang A.C., Orringer M.B., Pickens A., Pan C.C., Merajver S.D., Urba S.G. (2013). Pre-operative chemoradiation followed by post-operative adjuvant therapy with tetrathiomolybdate, a novel copper chelator, for patients with resectable esophageal cancer. Invest. New Drugs.

[49] Brewer G.J., Merajver S.D. (2002). Cancer therapy with tetrathiomolybdate: antiangiogenesis by lowering body copper—A review. Integr. Cancer Ther..

[50] Khan G., Merajver S. (2009). Copper chelation in cancer therapy using tetrathiomolybdate: an evolving paradigm. Expert Opin. Investig. Drugs.

[51] Sammons S., Brady D., Vahdat L., Salama A.K. (2016). Copper suppression as cancer therapy: The rationale for copper chelating agents in BRAF V600 mutated melanoma. Melanoma Manag..

[52] Brady D.C., Crowe M.S., Greenberg D.N., Counter C.M. (2017). Copper chelation inhibits BRAF V600E-driven melanomagenesis and counters resistance to BRAF V600E and MEK1/2 inhibitors. Cancer Res..

[53] Berg K.C.G., Eide P.W., Eilertsen I.A., Johannessen B., Bruun J., Danielsen S.A., Bjørnslett M., Meza-Zepeda L.A., Eknæs M., Lind G.E. (2017). Multi-omics of 34 colorectal cancer cell lines—A resource for biomedical studies. Mol. Cancer.

[54] Furukawa T., Komatsu M., Ikeda R., Tsujikawa K., Akiyama S. (2008). Copper transport systems are involved in multidrug resistance and drug transport. Curr. Med. Chem..

[55] Manzano J.L., Layos L., Bugés C., de Los Llanos Gil M., Vila L., Martínez-Balibrea E., Martínez-Cardús A. (2016). Resistant mechanisms to BRAF inhibitors in melanoma. Ann. Transl. Med..

[56] Faghfuri E., Nikfar S., Niaz K., Faramarzi M.A., Abdollahi M. (2018). Mitogen-activated protein kinase (MEK) inhibitors to treat melanoma alone or in combination with other kinase inhibitors. Expert Opin. Drug Metab. Toxicol..

[57] Ahmed D., Eide P.W., Eilertsen I.A., Danielsen S.A., Eknæs M., Hektoen M., Lind G.E., Lothe R.A. (2013). Epigenetic and genetic features of 24 colon cancer cell lines. Oncogenesis.

[58] Sini M.C., Doneddu V., Paliogiannis P., Casula M., Colombino M., Manca A., Botti G., Ascierto P.A., Lissia A., Cossu A. (2018). Genetic alterations in main candidate genes during melanoma progression. Oncotarget.

[59] Cosmic Cell Lines Project HCT-116 Mutations Cell Line Synopsis. http://www.webcitation.org/788mDTpZ8.

[60] Cosmic Cell Lines Project HT-29 Mutations Cell Line Synopsis. http://www.webcitation.org/788l6jCw9.

[61] Cosmic Cell Lines Project A-375 Mutations Cell Line Synopsis. http://www.webcitation.org/788on5GTQ.

[62] Guzmán C., Bagga M., Kaur A., Westermarck J., Abankwa D. (2014). ColonyArea: An ImageJ plugin to automatically quantify colony formation in clonogenic assays. PLoS ONE.

[63] Panzini G., Lorenzini R.N. (2004). Animal experimentation in Italy. Legislation and the authorization of research protocols. Ann Ist Super Sanita.

[64] Cox C., Merajver S.D., Yoo S., Dick R.D., Brewer G.J., Lee J.S., Teknos T.N. (2003). Inhibition of the growth of squamous cell carcinoma by tetrathiomolybdate-induced copper suppression in a murine model. Arch. Otolaryngol. Head Neck Surg..

[65] Xu M., Casio M., Range D.E., Sosa J.A., Counter C.M. (2018). Copper chelation as targeted therapy in a mouse model of oncogenic BRAF-driven papillary thyroid cancer. Clin. Cancer Res..

[66] Di Rocco G., Verdina A., Gatti V., Virdia I., Toietta G., Todaro M., Stassi G., Soddu S. (2016). Apoptosis induced by a HIPK2 full-length-specific siRNA is due to off-target effects rather than prevalence of HIPK2-Delta e8 isoform. Oncotarget.

[67] Di Rocco G., Gentile A., Antonini A., Truffa S., Piaggio G., Capogrossi M., Toietta G. (2012). Analysis of biodistribution and engraftment into the liver of genetically modified mesenchymal stromal cells derived from adipose tissue. Cell Transplant..

